# Impact of preoperative triglyceride levels on hepatic encephalopathy development in TIPS patients: a retrospective cohort study

**DOI:** 10.1186/s12876-025-04583-7

**Published:** 2026-01-12

**Authors:** Fei Cai, Jing Yu, Lei Qin

**Affiliations:** Department of Interventional Radiology, The First College of Clinical Medical Science, China Three Gorges University, Yichang Central People’s Hospital, Yichang, Hubei China

**Keywords:** Triglycerides, TIPS, Hepatic encephalopathy, Liver cirrhosis, Predictive marker

## Abstract

**Background:**

Overt hepatic encephalopathy (OHE) is a common complication following transjugular intrahepatic portosystemic shunt (TIPS) procedures in patients with cirrhosis.

However, the relationship between preoperative triglyceride (TG) levels and post-TIPS OHE risk remains unclear. This study aimed to investigate whether elevated preoperative TG levels are independently associated with increased risk of developing OHE following TIPS in patients with cirrhosis.

**Methods:**

This retrospective cohort study included 172 patients with cirrhosis who underwent TIPS at Yichang Central People's Hospital from January 2021 to December 2023. The primary exposure variable was preoperative TG level, and the primary outcome was the occurrence of OHE during follow-up. Cox proportional hazards regression models were used to analyze the relationship between TG levels and OHE risk, with stratified analyses conducted according to Child-Pugh classification and blood ammonia levels.

**Results:**

The incidence of OHE was significantly higher in the high TG group compared to the low TG group (45.2% vs 26.1%, P=0.026). After adjusting for multiple confounding factors, each 1 mmol/L increase in TG was associated with a 15.4% increase in OHE risk (HR=1.154, 95% CI: 1.042-1.426, P=0.036). Compared to the lowest quartile TG group, the highest quartile group showed a 26.5% increase in OHE risk (HR=1.265, 95% CI: 1.201-1.461, P=0.001). This association was more pronounced in patients with Child-Pugh class B/C.

**Conclusions:**

Elevated preoperative TG levels are independently associated with increased risk of post-TIPS OHE development. Preoperative TG level assessment may help identify high-risk patients and facilitate the development of appropriate preventive strategies.

## Introduction

 TIPS serves as a crucial intervention for treating complications of portal hypertension in liver cirrhosis [1, 2], yet its clinical application faces significant challenges. Post-procedural overt hepatic encephalopathy (OHE) represents one of the most common complications [3], with a global incidence rate reaching 35%−50% [4, 5], predominantly occurring within 1–3 months post-procedure [6]. 8% of patients develop refractory hepatic encephalopathy [7], a complication that not only severely compromises patient quality of life and prognosis but is also associated with elevated mortality rates [8], thereby constituting one of the primary barriers limiting the widespread application of TIPS [9].

The liver serves as the central hub for lipid metabolism, orchestrating the entire process of triglyceride synthesis, transport, and degradation [10, 11]. In patients with liver cirrhosis, as hepatic dysfunction progressively worsens, lipid metabolic disorders become increasingly pronounced [12]. Recent research demonstrates that serum lipid levels can serve as independent predictive factors for assessing declining hepatic functional reserve in patients with cirrhosis [13]. This lipid metabolic abnormality is not merely a consequence of hepatic dysfunction but is also closely associated with multiple cirrhotic complications, including sarcopenia, mysteatosis, and insulin resistance [14]. Notably, these complications have all been confirmed to correlate with increased risk of hepatic encephalopathy [15]. Furthermore, hypertriglyceridemia may induce or exacerbate systemic inflammatory responses, enhance oxidative stress levels, and lead to intestinal microbiota dysbiosis [10]. These pathophysiological changes may all constitute important mechanisms promoting the occurrence and progression of hepatic encephalopathy [15]. Therefore, preoperative triglyceride level abnormalities may influence the risk of postoperative hepatic encephalopathy through multiple pathways.

Despite the theoretical connection between lipid metabolism and the pathogenesis of hepatic encephalopathy, research on the relationship between preoperative triglyceride levels and the risk of post-TIPS hepatic encephalopathy remains limited. Previous studies have primarily focused on traditional risk factors such as age, blood ammonia levels, and hepatic functional reserve, while the potential predictive value of lipid metabolism parameters has been largely overlooked. To address this significant knowledge gap, we conducted a retrospective cohort study to systematically evaluate the correlation between preoperative triglyceride levels in patients with cirrhosis and the risk of hepatic encephalopathy within one year after TIPS. To our knowledge, no previous studies have systematically investigated the dose-response relationship between triglyceride levels and post-TIPS OHE, or tested this association across different patient subgroups. This study aims to provide new biochemical indicator references for preoperative risk stratification assessment of TIPS procedures, optimize postoperative complication monitoring and management strategies, and establish a theoretical foundation for hepatic encephalopathy prevention and intervention measures, ultimately improving the long-term survival rate and quality of life for patients undergoing TIPS.

## Methods

### Study population

**Fig. 1:**
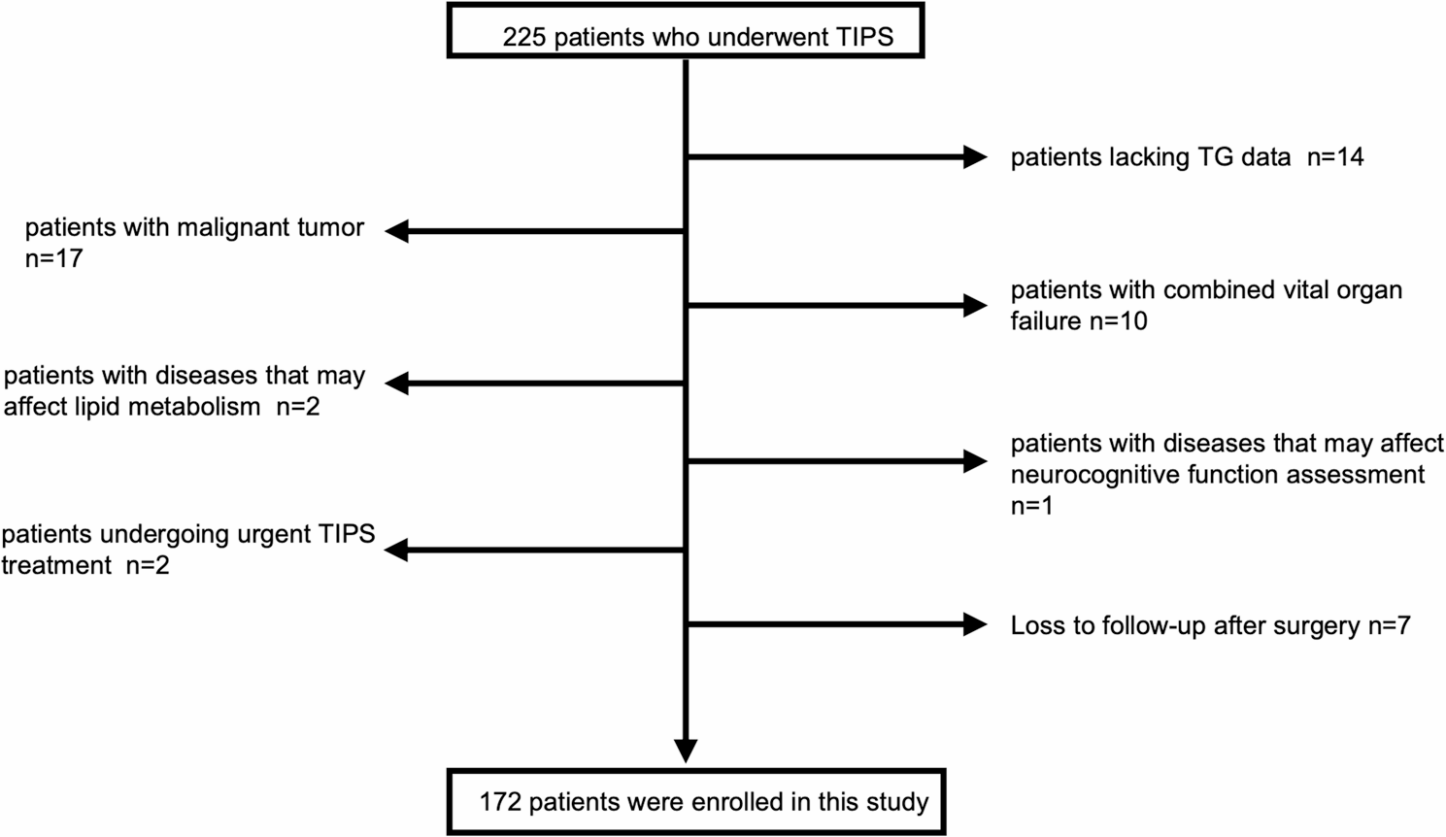
Patient Screening Flowchart

This was a retrospective cohort study conducted at Yichang Central People’s Hospital from January 1, 2021 to December 31, 2023, with follow-up extending until December 31, 2024. The study subjects comprised 172 patients with liver cirrhosis who underwent TIPS procedures. All patients were diagnosed with liver cirrhosis through medical history, laboratory, and imaging examinations. Inclusion criteria included: (1) age ≥ 18 years and ≤ 75 years; (2) clinically, laboratory, and imaging-confirmed diagnosis of liver cirrhosis with TIPS procedure; (3) complete preoperative testing records for lipid metabolism parameters including triglycerides; (4) post-TIPS follow-up of at least one year with documented records of hepatic encephalopathy occurrence. Exclusion criteria included: (1) preoperative confirmed diagnosis or clear history of hepatic encephalopathy; (2) preoperative confirmed diagnosis of acute-on-chronic liver failure; (3) complete portal vein occlusion requiring portal vein recanalization; (4) concurrent active hepatocellular carcinoma or other malignancies; (5) concurrent severe dysfunction of vital organs including heart, lung, or kidney; (6) concurrent diseases affecting neurocognitive function assessment (such as Alzheimer’s disease, Parkinson’s disease, etc.); (7) concurrent specific diseases affecting lipid metabolism (such as severe thyroid dysfunction, uncontrolled diabetes, etc.); (8) recent treatment with specific lipid-regulating medications (within 3 months preoperatively); (9) emergency TIPS treatment (such as within 72 h following acute variceal bleeding); (10) liver transplantation before surgery or during follow-up period; (11) severely incomplete clinical data or loss to follow-up within one year postoperatively. The flow diagram with information on excluded patients is detailed in Fig. 1. Patient data were collected through the hospital’s electronic medical record system, including demographic characteristics, clinical biochemical examinations, imaging studies, TIPS procedure-related information, and follow-up records.

This flowchart illustrates the systematic patient screening process of this study. Among 225 patients who underwent transjugular intrahepatic portosystemic shunt (TIPS) treatment at Yichang Central People’s Hospital between January 1, 2021, and December 31, 2023, 53 patients were excluded based on the following criteria: 14 patients lacked preoperative triglyceride (TG) data, 17 patients had concomitant malignant tumors, 10 patients had major organ failure, 2 patients had diseases that could potentially affect lipid metabolism, 1 patient had a disease that could potentially affect neurocognitive function assessment, 2 patients received emergency TIPS treatment, and 7 patients were lost to follow-up postoperatively. The final study population comprised 172 patients who met all inclusion criteria and completed the 12-month follow-up protocol or were followed until the occurrence of overt hepatic encephalopathy (OHE).

### Variable

#### Exposure variable

The exposure variable in this study was preoperative triglyceride levels. Serum triglycerides were measured from fasting venous blood samples collected within 24 h prior to TIPS procedure. All samples were processed and analyzed by the hospital laboratory department according to standard operating procedures to ensure measurement accuracy and consistency. Triglyceride levels were recorded in the database as continuous variables (mmol/L).

#### Outcome variable

The primary outcome variable was the occurrence of hepatic encephalopathy within one year following TIPS procedure. Hepatic encephalopathy (HE) was diagnosed and graded according to the West Haven criteria, in accordance with the 2014 AASLD/EASL clinical practice guideline (Vilstrup et al., Hepatology 2014) [2].Clinical assessment was independently completed by two experienced hepatology specialists, and in case of disagreement, a third senior expert made the final determination. Since this was a retrospective study, outcome assessment was not conducted in a blinded manner. The follow-up protocol consisted of monthly visits during the first 1–3 months post-TIPS, followed by visits every 3 months from 3 to 12 months post-procedure. The outcome variables were recorded in the database as binary variables (yes/no) and time to first occurrence (days post-procedure).

### Covariate

The included covariates comprised: (1) demographic characteristics: age and sex; (2) clinical characteristics: TIPS indication (esophagogastric variceal bleeding, ascites, or both), etiology of cirrhosis (viral, alcohol-related, biliary, or other); (3) liver function-related parameters: Child-Pugh score; (4) other laboratory parameters: blood ammonia, creatinine, hemoglobin, and C-reactive protein; (5) surgery-related factors: preoperative hepatic venous pressure gradient (HVPG) and portal vein shunt branch selection (left branch, right branch, or other). These variables were selected as covariates based on previous studies demonstrating their association with post-TIPS hepatic encephalopathy development. Among these, Child-Pugh scores reflect the degree of hepatic functional impairment, elevated blood ammonia levels are considered an independent risk factor for hepatic encephalopathy, and surgery-related parameters may influence postoperative complication risk.

### TIPS procedure

All TIPS procedures were performed using Viatorr covered stent-grafts with a uniform diameter of 8 mm (Gore Viatorr; W.L. Gore & Associates, Flagstaff, AZ, USA). Procedures were conducted by experienced interventional radiologists under fluoroscopic guidance following standard techniques. The standardized stent diameter was used for all patients to ensure procedural consistency and minimize variability.

### Statistical analysis

Continuous variables were expressed as mean ± standard deviation (SD) or median (interquartile range, IQR) according to their distribution characteristics, while categorical variables were presented as frequencies and percentages. For between-group comparisons, categorical variables were analyzed using χ² test, and continuous variables were analyzed using Student’s t-test (for normal distribution) or Mann-Whitney U test (for skewed distribution) based on their distribution properties. Fisher’s exact test was employed when the expected frequency in any cell was less than 5. The association between serum triglyceride (TG) levels and the risk of overt hepatic encephalopathy (OHE) was evaluated using Cox proportional hazards regression models, calculating hazard ratios (HR) and 95% confidence intervals (CI). In the analysis, TG was examined both as a continuous variable and as a categorical variable grouped by quartiles (Q1-Q4). Three models with different adjustment levels were constructed: an unadjusted model, a minimally adjusted model (adjusted for age and sex), and a fully adjusted model (adjusted for multiple clinical variables). Linear trend tests were performed by incorporating the median values of each TG quartile group as continuous variables in the regression model. To explore potential heterogeneity in this association, we conducted stratified analyses based on key clinical variables, including Child-Pugh score, blood ammonia levels and TIPS indications. Fully adjusted hazard ratios and corresponding P-values were calculated within each subgroup. Effect modification was assessed by evaluating interactions between TG levels and stratifying variables through likelihood ratio tests. During the 12-month follow-up period, no deaths occurred among study participants; therefore, Cox proportional hazards regression was used as the appropriate analytical method without requiring competing risk analysis.All statistical analyses used *P* < 0.05 as the significance level.

## Results

### Patient general characteristics

All 172 patients were successfully followed for the entire 12-month period or until OHE development. They were divided into two groups based on serum triglyceride (TG) levels: 88 patients with TG ≤ 1.5 mmol/L (low TG group) and 84 patients with TG > 1.5 mmol/L (high TG group). The two groups showed no statistically significant differences in age (58.0±12.2 years vs 60.0±10.5 years, P=0.658), gender distribution (male: 64.8% vs 65.5%, P=0.721), TIPS procedure indications (P=0.052), etiology of cirrhosis (P=0.561), Child-Pugh score (7.0 [6.0, 9.0] vs 8.0 [7.0, 10.0], P=0.290). However, significant differences were observed between the two groups in blood ammonia levels (44.7±14.8 vs 52.4±20.4 μmol/L, P=0.036) and C-reactive protein levels (2.5 [0.5, 12.2] vs 5.7 [3.9, 47.6] mg/L, P=0.029), with patients in the high TG group showing significantly elevated levels. Regarding surgical details, the preoperative hepatic venous pressure gradient was similar between the two groups (18.0 [14.0, 21.0] vs 20.0 [12.0, 23.0] mmHg, P=0.280). However, there was a significant difference in the distribution of portal vein shunt branches (P=0.013), with the high TG group having a higher proportion of right branch shunts (35.7% vs 28.4%) and a lower proportion of left branch shunts (46.4% vs 56.8%). During follow-up, the incidence of overt hepatic encephalopathy (OHE) was significantly higher in patients with TG > 1.5 mmol/L (45.2% vs 26.1%, P=0.026), as detailed in Table 1.

**Table 1:** Baseline demographic, clinical, and laboratory characteristics of patients with cirrhosis stratified by preoperative triglyceride levels

Characteristic	TG ≤ 1.5 mmol/L (*n* = 88)	TG > 1.5 mmol/L (*n* = 84)	*P* Value	
Demographics				
Age, mean ± SD, years	58.0 ± 12.2	60.0 ± 10.5		0.658
Sex, n (%)				0.721
Male	57(64.8)	55(65.5)		
Female	31(35.2)	29(34.5)		
Clinical Parameters				
TIPS indication, n (%)				0.052
Variceal bleeding	72(81.8)	65(77.4)		
Ascites	13(14.8)	14(16.7)		
Bleeding and ascites	3(3.4)	5(5.9)		
Cirrhotic etiology, n (%)				0.561
Viral	52(59.1)	46(54.8)		
Alcohol-associated	20(22.7)	21(25.0)		
Biliary	9(10.2)	8(9.5)		
Other	7(8.0)	9(10.7)		
Child-Pugh score, median (IQR)	7.0 (6.0, 9.0)	8.0 (7.0, 10.0)		0.290
Laboratory Values				
Blood ammonia, mean ± SD, µmol/L	44.7 ± 14.8	52.4 ± 20.4		0.036
Creatinine, median (IQR), µmol/L	66.0 (54.0, 77.0)	70.0 (59.0, 85.0)		0.084
Hb, mean ± SD, g/L	82.0 ± 23.5	86.0 ± 27.1		0.972
CRP, median (IQR), mg/L	2.5 (0.5, 12.2)	5.7 (3.9, 47.6)		0.029
Procedural Details				
Preoperative HVPG, median (IQR), mmHg	18.0 (14.0, 21.0)	20.0 (12.0, 23.0)		0.280
Shunting branch of PV, n (%)				0.013
Left	50 (56.8)	39 (46.4)		
Right	25 (28.4)	30 (35.7)		
Other	13 (14.8)	15 (17.9)		
Outcome				
OHE during follow-up, n (%)				0.026
Yes	23 (26.1)	38 (45.2)		
No	65 (73.9)	46 (54.8)		

*CRP* C-reactive protein, *Hb* Hemoglobin, *HVPG* Hepatic venous pressure gradient, *IQR* Interquartile range, *OHE* Overt hepatic encephalopathy, *PV* Portal vein, *SD* Standard deviation, *TG* Triglycerides, *TIPS* Transjugular intrahepatic portosystemic shunt

P-value <0.05 considered statistically significant

### Relationship between serum TG levels and hepatic encephalopathy after TIPS

We conducted multilevel analyses using Cox proportional hazards regression models (Table 2), including crude model, minimally adjusted model (adjusted for age and sex), and fully adjusted model (adjusted for age, sex, TIPS indication, cirrhotic etiology, Child-Pugh score, blood ammonia, creatinine, hemoglobin, C-reactive protein, preoperative HVPG, and shunting branch of portal vein). When TG was analyzed as a continuous variable, the crude model showed that each 1 mmol/L increase in TG was associated with a 43.8% increased risk of OHE (HR = 1.438, 95%CI 1.273–1.785, P = 0.001). After adjusting for age and sex, this association was slightly attenuated but remained significant (HR = 1.394, 95%CI 1.180–1.560, P = 0.026). In the fully adjusted model, even after controlling for traditional liver disease severity scores and known metabolic risk factors, TG retained independent predictive value, with each 1 mmol/L increase conferring a 15.4% increased risk of OHE (HR = 1.154, 95%CI 1.042–1.426, P = 0.036). To further explore the association pattern between TG levels and OHE risk, TG was categorized into quartiles for analysis. The crude model demonstrated a clear stepwise effect, with progressively increasing OHE risk in the second, third, and fourth quartiles compared with the lowest quartile (Q1) (Q2: HR = 1.251, P = 0.004; Q3: HR = 1.447, P = 0.001; Q4: HR = 1.623, P = 0.001; P for trend < 0.001). In the fully adjusted model, the risk increase remained statistically significant across all elevated TG groups, with Q2, Q3, and Q4 showing 18.4% (HR = 1.184, 95%CI 1.020–1.360, P = 0.031), 22.4% (HR = 1.224, 95%CI 1.137–1.420, P = 0.015), and 26.5% (HR = 1.265, 95%CI 1.201–1.461, P = 0.001) increased OHE risk compared with Q1, respectively. Importantly, the trend test remained statistically significant in the fully adjusted model (P = 0.006), confirming an independent dose-response relationship between TG levels and OHE risk.

**Table 2:** Relationship between TG and overt hepatic encephalopathy

Variable	Crude model	Minimally adjusted model	Fully adjusted model
TG	1.438 (1.273, 1.785) 0.001	1.394 (1.180, 1.560) 0.026	1.154 (1.042, 1.426) 0.036
TG (quartile)			
Q1	Ref	Ref	Ref
Q2	1.251 (1.017, 1.584) 0.004	1.227 (1.120, 1.520) 0.002	1.184 (1.020, 1.360) 0.031
Q3	1.447 (1.241, 1.728) 0.001	1.406 (1.382, 1.553) 0.001	1.224 (1.137, 1.420) 0.015
Q4	1.623 (1.284, 1.822) 0.001	1.526 (1.241, 1.739) 0.001	1.265 (1.201, 1.461) 0.001
P for trend	<0.001	<0.001	0.006

Crude model:we did not adjust other covariants

Minimally adjusted model:we adjusted sex and age

Fully adjusted model:we adjusted age, sex, TIPS indication, Cirrhotic etiology, Child-Pugh score, Blood ammonia, Creatinine Hb, CRP, Preoperative HVPG, Shunting branch of PV

*CI* Confidence interval, *Ref* Reference

### Stratified analysis of the association between triglyceride levels and Post-TIPS OHE risk

To evaluate whether the predictive value of TG for OHE risk differs across populations with different clinical characteristics, we conducted subgroup analyses and tested for interactions (Table 3). After stratification by Child-Pugh score, the association between TG and OHE risk was significant across all liver function grades, but the effect sizes showed a clear gradient of increasing magnitude: in Child-Pugh class A patients, each 1 mmol/L increase in TG was associated with a 28.0% increase in OHE risk (HR = 1.280, 95%CI 1.050–1.567, *P* = 0.045), whereas in class B and C patients, this risk increment reached 35.4% (HR = 1.354, 95%CI 1.154–1.675, *P* = 0.018) and 41.3% (HR = 1.413, 95%CI 1.187–1.725, *P* = 0.008), respectively. The interaction test showed statistical significance (P for interaction = 0.012), indicating that the effect of TG on OHE intensifies with deteriorating liver function. Stratified analysis by blood ammonia levels revealed that the impact of TG on OHE risk remained consistent regardless of whether baseline blood ammonia was elevated, with HRs of 1.208 (95%CI 1.182–1.871, *P* = 0.035) and 1.243 (95%CI 1.047–1.842, *P* = 0.015) for the normal and elevated ammonia groups, respectively, and the interaction test was not statistically significant (P for interaction = 0.180). After stratification by TIPS indication, the strength of association between TG and OHE risk was similar in patients who received TIPS treatment for either variceal bleeding (HR = 1.301, 95%CI 1.182–1.624, *P* = 0.006) or refractory ascites (HR = 1.252, 95%CI 1.075–1.846, *P* = 0.028), with no statistical significance in the interaction test (P for interaction = 0.642).

**Table 3:** Stratified analysis of the association between serum triglyceride levels and overt hepatic encephalopathy risk after TIPS

Stratification Variable	Subgroup	HR (95% CI)	*P* value	*P* for interaction
Child-Pugh score	A	1.280 (1.050–1.567)	0.045	0.012
	B	1.354 (1.154–1.675)	0.018	
	C	1.413 (1.187–1.725)	0.008	
Blood ammonia	Normal	1.208 (1.182–1.871)	0.035	0.180
	Elevated	1.243 (1.047–1.842)	0.015	
TIPS indication	Variceal bleeding	1.301 (1.182–1.624)	0.006	0.642
	Ascites	1.252 (1.075–1.846)	0.028	

*TIPS* Transjugular intrahepatic portosystemic shunt, *HR *Hazard ratio, *CI *Confidence interval

## Discussion

In this retrospective cohort study involving 172 patients who underwent TIPS, we investigated the association between preoperative serum triglyceride levels and the occurrence of overt hepatic encephalopathy (OHE) within one year post-procedure. The strengths of this study lie in comprehensive clinical data collection, detailed laboratory assessment, and systematic stratified analysis of key clinical parameters. The results demonstrate that elevated preoperative triglyceride levels are independently associated with increased risk of hepatic encephalopathy following TIPS: for each 1 mmol/L increase in triglycerides, the risk of OHE increases by 15.4% (HR=1.154, 95% CI: 1.042–1.426.042.426, P=0.036). More notably, compared to patients in the lowest triglyceride quartile, those in the highest quartile showed a 26.5% increased risk of OHE (HR=1.265, 95% CI: 1.201–1.461.201.461, P=0.001), with a significant dose-response relationship observed with increasing triglyceride levels (P for trend=0.006).

This study found that elevated preoperative serum triglyceride levels are significantly associated with increased risk of overt hepatic encephalopathy after TIPS, consistent with multiple studies exploring the relationship between lipid metabolism and hepatic encephalopathy. Notably, Taniguchi et al.'s cross-sectional study of 88 patients with viral cirrhosis demonstrated that elevated free fatty acids and triglycerides were independently associated with cognitive dysfunction in patients with cirrhosis [16]. They not only identified lipid profiles as potential biomarkers for early detection of hepatic encephalopathy but also quantified that elevated triglycerides significantly increased the risk of cognitive impairment (OR = 1.15, 95% CI: 1.02–1.29). Similarly, Gu Lihong et al.‘s research supported our findings through a retrospective cohort study of 145 patients with cirrhosis undergoing TIPS [17], which showed that overweight/obesity was an independent risk factor for postoperative overt hepatic encephalopathy (OHE). In their study, 52 (35.9%) overweight/obese patients had significantly higher postoperative OHE incidence compared to normal-weight patients (OR = 2.754, 95% CI: 1.236–6.140; *p* = 0.013), and Kaplan-Meier survival curve analysis showed the highest cumulative incidence of OHE in overweight/obese patients (log-rank *p* = 0.0118). This finding complements our triglyceride results, as lipid metabolism disorders and abnormal body fat distribution are closely associated with overweight/obesity and collectively influence the mechanisms of hepatic encephalopathy. However, Nardelli et al.‘s study failed to confirm a significant association between serum triglyceride levels and hepatic encephalopathy risk [18]. In their observations of patients with alcoholic cirrhosis, serum cholesterol levels, rather than triglycerides, were primarily associated with hepatic encephalopathy occurrence. This discrepancy in research results may be attributed to multiple factors: first, significant differences in study population characteristics, as Nardelli et al.‘s cohort primarily included patients with alcoholic cirrhosis whose baseline metabolic characteristics differed from our study subjects; second, differences in measurement standards and definition methods for exposure and outcome variables between the two studies; additionally, regarding statistical analysis, Nardelli et al.‘s study adjusted for fewer covariates and used simpler statistical models, potentially failing to adequately control for potential confounding factors, thereby affecting the reliability of their conclusions. From a pathophysiological perspective, elevated triglycerides may promote hepatic encephalopathy development through multiple interconnected pathways. First, hypertriglyceridemia reflects lipid metabolic dysregulation and exacerbates systemic inflammation and endotoxemia through TLR4/NF-κB signaling [19], which synergizes with hyperammonemia to promote blood-brain barrier disruption and neuroinflammation—key elements in the pathogenesis of hepatic encephalopathy [20]. Second, lipid metabolism abnormalities impair astrocyte mitochondrial function, reducing their capacity to detoxify ammonia through the glutamine synthetase pathway [21, 22], thereby promoting cerebral edema and neurological dysfunction [23]. Additionally, elevated TG is associated with gut dysbiosis, increasing intestinal ammonia production and bacterial translocation, which synergistically amplifies neurotoxic substrate delivery to the brain in patients undergoing TIPS [24]. Furthermore, elevated TG in patients with cirrhosis may reflect impaired hepatic VLDL synthesis and biosynthetic capacity, indicating reduced metabolic reserve to handle TIPS-induced ammonia burden—a dimension not fully captured by traditional liver function scores [25]. Finally, oxidative stress generated by hypertriglyceridemia promotes cerebrovascular endothelial dysfunction and impairs cerebral blood flow autoregulation, potentially enhancing neuronal vulnerability to ammonia toxicity [26]. Notably, the observed dose-response relationship (P for trend = 0.006) and the significantly stronger TG effect in Child-Pugh B/C patients (P for interaction = 0.012) further support the biological plausibility of these mechanisms, suggesting synergistic interaction between TG-mediated metabolic stress and reduced hepatic detoxification capacity.

This research systematically elucidates the significant association between pre-procedural triglyceride levels and the risk of post-TIPS hepatic encephalopathy, which has important clinical value. Our findings indicate that elevated pre-procedural triglyceride levels (particularly ≥1.5 mmol/L) represent an independent risk factor for post-TIPS hepatic encephalopathy, with a clear dose-response relationship between the two. While existing studies have primarily focused on traditional predictive factors such as ammonia levels, age, and liver function status, they have overlooked the potential impact of lipid metabolism abnormalities [27–29]. This study fills this gap, providing a new perspective for risk assessment. In clinical practice, we recommend incorporating serum triglyceride levels into the pre-TIPS evaluation system, with particular emphasis on screening patients with Child-Pugh class B/C, as the association between triglycerides and hepatic encephalopathy is especially significant in these subgroups. Furthermore, for high-risk patients identified with elevated triglyceride levels, individualized intervention strategies should be considered, such as pre-procedural lipid-regulating therapy, optimization of stent selection during the procedure, or enhanced post-procedural monitoring protocols. Future research may further explore the development of combined predictive models using pre-procedural triglyceride levels with other lipid metabolism indicators, as well as the long-term impact of dynamic changes in triglyceride levels on the occurrence and prognosis of hepatic encephalopathy, providing a more solid theoretical foundation for developing targeted prevention and treatment strategies.

This study has the following limitations. First, covert hepatic encephalopathy was not systematically evaluated before TIPS, and its absence may affect the accuracy of predictions. Second, as a single-center retrospective study, the generalizability of the findings is limited, and future multicenter studies are needed to further validate the findings of this study. Third, this study excluded patients with severe cardiovascular and cerebrovascular diseases and malignant tumors; therefore, the results are not applicable to these special populations. Fourth, as an observational study, we can only reveal the association between triglyceride levels and post-TIPS hepatic encephalopathy, but cannot establish causality. Fifth, the precipitating factors for each episode of hepatic encephalopathy (such as infection, gastrointestinal bleeding, or poor medication compliance) were not systematically recorded, which limits our ability to distinguish between spontaneous and precipitated events. Furthermore, although we adjusted for known confounding factors in the statistical analysis, the potential influence of unmeasured or unknown confounders cannot be excluded.

## Conclusions

In conclusion, preoperative serum triglyceride levels are closely associated with the occurrence of hepatic encephalopathy following TIPS procedure and can serve as a predictive biomarker for post-procedural hepatic encephalopathy. Detection of triglyceride levels prior to TIPS in patients with liver cirrhosis will facilitate the identification of high-risk patients and enable the development of appropriate treatment and prevention strategies.

## Data Availability

The datasets generated during and/or analyzed during the current study are available from the corresponding author on reasonable request.
